# Long-term clinical outcomes of image-guided percutaneous coronary intervention in acute myocardial infarction from the Korea Acute Myocardial Infarction Registry

**DOI:** 10.1371/journal.pone.0304843

**Published:** 2024-06-05

**Authors:** Youngjoon Kwon, Namkyun Kim, Chang-Yeon Kim, Do-Hoon Kim, Hyewon Shin, Min-Su Jung, Jong Sung Park, Yoon Jung Park, Bo Eun Park, Hong Nyun Kim, Se Yong Jang, Myung Hwan Bae, Jang Hoon Lee, Dong Heon Yang, Hun Sik Park, Yongkeun Cho, Kwang Soo Cha, Seung-Ho Hur, Jin-Yong Hwang, Myung Ho Jeong, on behalf of the KAMIR- NIH (Korea Acute Myocardial Infarction Registry- National Institutes of Health) Investigators

**Affiliations:** 1 Department of Internal Medicine, Kyungpook National University Hospital, Daegu, Republic of Korea; 2 School of Medicine, Kyungpook National University, Daegu, Republic of Korea; 3 Department of Internal Medicine, School of Medicine, Daegu Catholic University Medical Center, Daegu, Republic of Korea; 4 Department of Nuclear Medicine, Daejeon Eulji Medical Center, Eulji University School of Medicine, Daejeon, Republic of Korea; 5 Department of Biostatistics, College of Medicine, Korea University, Seoul, Republic of Korea; 6 Division of Biostatistics, Linical Korea Co., Ltd, Seoul, Republic of Korea; 7 Pusan National University Hospital, Busan, Republic of Korea; 8 Keimyung University Dongsan Medical Center, Cardiovascular Medicine, Deagu, Republic of Korea; 9 Department of Internal Medicine, Gyeonsang National University School of Medicine, Gyeongsang National University Hospital, Jinju, Republic of Korea; 10 Chonnam National University Hospital, Gwangju, Republic of Korea; East Tennessee State University, UNITED STATES

## Abstract

Imaging modalities for percutaneous coronary intervention (PCI), such as intravascular ultrasound (IVUS) or optical coherence tomography (OCT), have increased in the current PCI era. However, their clinical benefits in acute myocardial infarction (AMI) have not been fully elucidated. This study investigated the long-term outcomes of image-guided PCI in patients with AMI using data from the Korean Acute Myocardial Infarction Registry. A total of 9,271 patients with AMI, who underwent PCI with second-generation drug-eluting stents between November 2011 and December 2015, were retrospectively examined, and target lesion failure (TLF) at 3 years (defined as the composite of cardiac death, target vessel myocardial infarction, and ischemia-driven target lesion revascularization) was evaluated. From the registry, 2,134 patients (23.0%) underwent image-guided PCI (IVUS-guided: n = 1,919 [20.6%]; OCT-guided: n = 215 patients [2.3%]). Based on propensity score matching, image-guided PCI was associated with a significant reduction in TLF (hazard ratio: 0.76; 95% confidence interval: 0.59–0.98, p = 0.035). In addition, the TLF incidence in the OCT-guided PCI group was comparable to that in the IVUS-guided PCI group (5.3% vs 4.7%, p = 0.903). Image-guided PCI, including IVUS and OCT, is associated with favorable clinical outcomes in patients with AMI at 3 years post-intervention. Additionally, OCT-guided PCI is not inferior to IVUS-guided PCI in patients with AMI.

## Introduction

Image-guided percutaneous coronary intervention (PCI), using techniques such as intravascular ultrasound (IVUS) or optical coherence tomography (OCT), offers valuable clinical information for interventional cardiologists. The morphology and composition of coronary plaques can be visualized during preintervention evaluation. During stenting, the size and apposition of the stent are finely adjusted, and landing zones can be confirmed. Moreover, the extent of stent expansion, edge dissection, and hematoma can be identified in the postprocedural step [1–3]. Therefore, in high-risk patients or those with severe stenosis requiring complex interventions, IVUS- or OCT-guided PCI is particularly recommended by current practices [4,5].

Several studies have been conducted to deepen our understanding of the significance of image-guided PCI across various clinical scenarios [6,7]. Of note, the Randomized Controlled Trial of Intravascular Imaging Guidance versus Angiography-Guidance on Clinical Outcomes after Complex Percutaneous Coronary Intervention (RENOVATE-COMPLEX-PCI) trial demonstrated that image-guided PCI is associated with a lower risk of composite outcomes, including cardiac death, target vessel-related myocardial infarction, or clinically driven target vessel revascularization, compared with angiography-guided PCI alone [8].

Regarding different modalities for image-guided PCI, several studies have compared the outcomes of IVUS versus OCT. The recently published Optical Coherence Tomography-Guided or Intravascular Ultrasound-Guided Percutaneous Coronary Intervention trial established that OCT-guided PCI is noninferior to IVUS-guided PCI regarding the incidence of a composite of death from cardiac causes, target vessel-related myocardial infarction, or ischemia-driven target vessel revascularization at 1 year [7].

Nevertheless, the long-term clinical importance of image-guided PCI in acute myocardial infarction (AMI) remains incompletely understood, despite the increasing use of IVUS or OCT in clinical practice. Herein, we aimed to elucidate the long-term impact of image-guided PCI in patients with AMI using multicenter and nationwide registry data in the contemporary PCI era.

## Materials and methods

### Study design and population

**Fig 1:**
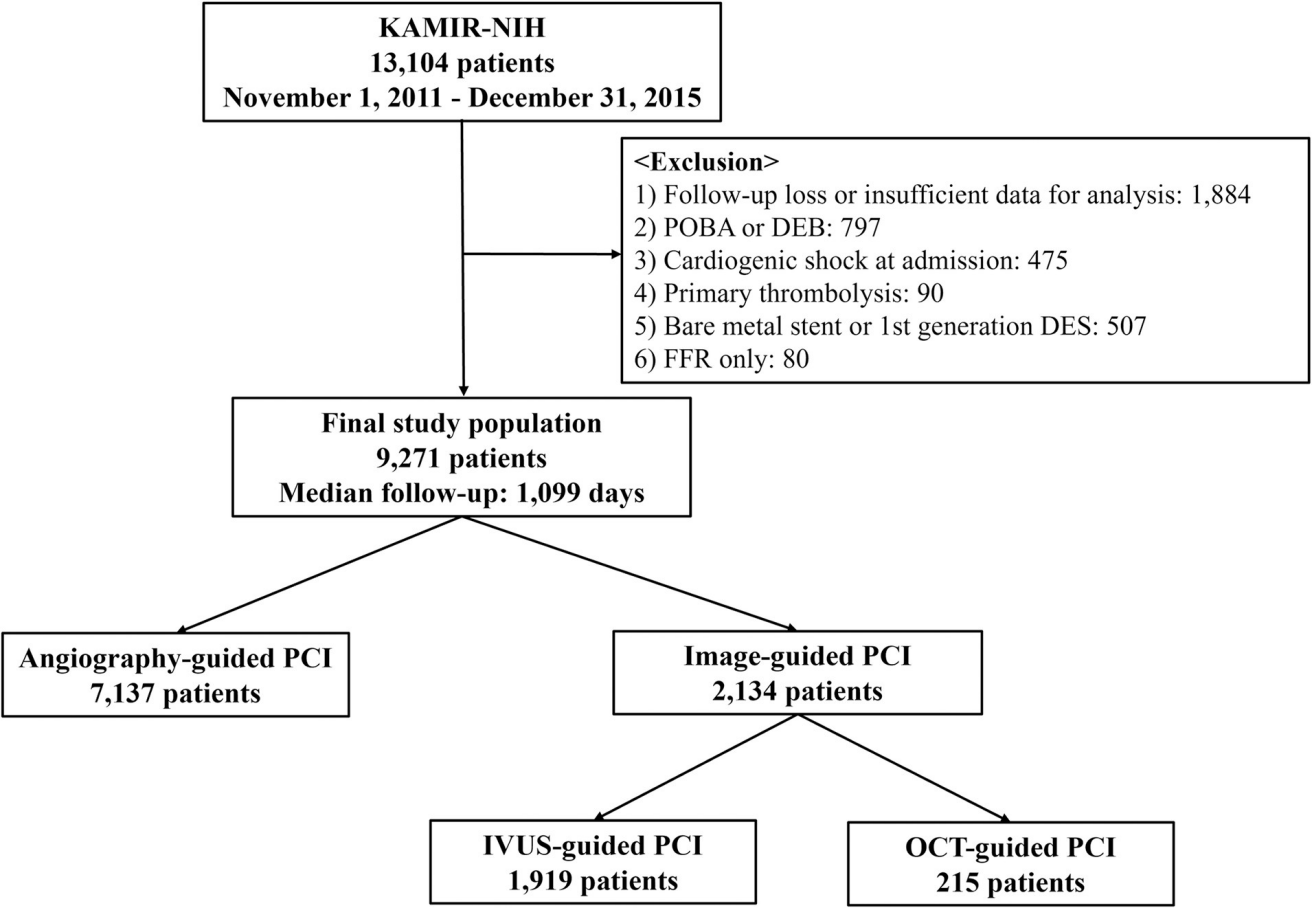
Study overview. KAMIR-NIH, Korea Acute Myocardial Infarction Registry—National Institutes of Health; IVUS, intravascular ultrasound; OCT, optical coherence tomography; PCI, percutaneous coronary intervention; FFR, fractional flow reserve; POBA, plain old balloon angioplasty; DEB, drug-eluting balloon; DES, drug-eluting stent.

A retrospective review of all patients registered in the Korean Acute Myocardial Infarction Registry—National Institutes of Health (KAMIR-NIH), who underwent PCI with second-generation drug-eluting stents (DES) between November 2011 and December 2015, was conducted. KAMIR-NIH is a prospective, open, observational, multicenter online registry of AMI cases in the Republic of Korea. A study overview is illustrated in Fig 1. This study was approved by the Institutional Review Board of Kyungpook National University Hospital (IRB No: KNUH-2022-01-011), and all patients provided written informed consent to participate. The registry data were accessed from January 15, 2023, to February 1, 2023, for the purposes of this research. Patients presenting with cardiogenic shock as their initial manifestation were excluded from this study. Those who received primary thrombolysis before undergoing PCI were also not included. Additionally, individuals assessed solely by fractional flow reserve (FFR) and those treated with bare-metal stents or first-generation DES were excluded. The final study population (n = 9,271) was stratified into two groups based on the modality used for PCI: image- (n = 2,134) and angiography-guided (n = 7,137). The image-guided PCI population was further classified into IVUS-guided (n = 1,919) and OCT-guided PCI (n = 215).

AMI, including both ST-segment elevation myocardial infarction (STEMI) and non-ST-segment elevation myocardial infarction (NSTEMI), was diagnosed according to the standard criteria of clinical manifestations and elevated serum cardiac troponin I levels [9]. All interventional procedures were performed according to current guidelines. The diagnostic modality (IVUS/OCT) was determined by the treating cardiologists in the catheterization laboratory.

IVUS or OCT was used to analyze the characteristics of plaque and target lesions during the preinterventional phase in the image-guided PCI group [10]. Additionally, the treating physicians used IVUS or OCT to obtain information on the landing zones, stent apposition and expansion, hematoma, or coronary dissection in the peri- and post-interventional phases [2].

In accordance with the current guidelines, all patients were administered aspirin and P2Y12 inhibitors. Prior to the procedure, each patient received a loading dose of aspirin (300 mg) and either clopidogrel (300 or 600 mg), prasugrel (60 mg), or ticagrelor (180 mg), depending on the practices of the individual centers. Specifically, 2,656 patients (28%) in the clopidogrel group were administered a 300mg loading dose. Administration of unfractionated heparin during PCI followed local practice rules and protocols. Patients received guideline-directed medical therapy, based on their individual conditions, and determined by the physician, which included the administration of antiplatelet agents, renin-angiotensin-system inhibitors, beta-blockers, and statins.

During initial admission, baseline patient information and laboratory data were collected, and subsequent data were obtained through medical records and direct telephone interviews. The outcome of the study was target lesion failure (TLF) at 3 years after the initial intervention, defined as the composite of all cardiac deaths, target vessel myocardial infarction, and ischemia-driven target lesion revascularization. Target vessel myocardial infarction is defined as a myocardial infarction marked by myocardial necrosis occurring in the vascular area served by a previously treated target vessel. Target lesion revascularization was classified as ischemia-driven if revascularization procedures were conducted on the target lesion under specific conditions: either in the case of ≥50% angiographic diameter stenosis accompanied by ischemic symptoms or a positive functional study, or when there was ≥70% stenosis without ischemic symptoms or a positive functional study.

### Statistical analysis

Continuous variables are presented as mean ± standard deviation (SD), whereas categorical variables are expressed as percentages. The comparison of parameters involved using the Student’s t-test for continuous variables and Pearson’s chi-square test for categorical variables. As the patient distribution was not uniform across study groups, propensity score (PS) matching was used to adjust for hidden confounding factors. PS was derived through logistic regression considering clinically important parameters between the two groups (age, sex, body mass index, clinical presentation, final diagnosis, hypertension, diabetes, dyslipidemia, previous myocardial infarction or cerebrovascular accident, left ventricular ejection fraction [LVEF], estimated glomerular filtration rate [eGFR], troponin I, creatine kinase-myocardial band [CK-MB], discharge medication, use of transradial approach, use of glycoprotein IIb/IIIa inhibitors, thrombus aspiration, stent diameter and length, stent type, stent number, number of vessels involved, multivessel disease, culprit vessel, and ACC/AHA lesion type). We used nearest-neighbor matching with a caliper size set at 0.2 times the SD of the logit-transformed PS.

We calculated the hazard ratios (HRs) and 95% confidence intervals (CI) from a matched stratified Cox proportional-hazards model for the main outcome in the PS-matched cohort. Cumulative clinical events at 3 years were estimated using the Kaplan–Meier survival analysis, and outcomes were compared using the log-rank test for each result. Statistical significance was set at p < 0.05. All analyses were conducted using R (version 4.3.1; R Foundation for Statistical Computing.).

## Results

### Baseline characteristics

Among the 9,271 patients retrieved from the registry, 2,134 (23.0%) underwent image-guided PCI; the median follow-up period of the entire population was 1,099 days. Within the image-guided PCI group, 1,919 patients (20.6%) underwent IVUS-guided PCI, and 215 patients (2.3%) underwent the OCT-guided PCI. The angiography-guided PCI group had a higher average patient age (63.29 ± 12.39 years vs 62.23 ± 12.10 years; p < 0.001) and a higher prevalence of previous hypertension (n = 3,598 [50.4%] vs n = 963 [45.1%]; p < 0.001) and cerebrovascular accidents (n = 434 [6.1%] vs n = 104 [4.9%]; p = 0.041) than the image-guided PCI group. Moreover, the angiography-guided PCI group exhibited significantly lower levels of LVEF on admission (52.12% ± 10.44% vs 53.14% ± 9.82%; p < 0.001).

However, the troponin I levels at admission were significantly higher in the angiography-guided PCI group than in the image-guided PCI group (47.02 ± 96.11 ng/mL vs 32.93 ± 118.25 ng/mL; p < 0.001). Furthermore, the frequency of RAS inhibitor usage was considerably higher in the angiography-guided PCI group (n = 5,981 [83.8%] vs n = 1,713 [80.3%]; p = 0.001), as was the use of beta-blockers (n = 6,259 [87.7%] vs 1,799 [84.3%]; p < 0.001).

Regarding procedural characteristics, the transradial approach was more commonly preferred in the image-guided PCI group (n = 920 [43.1%] versus n = 2,683 [37.6%]; p < 0.001). Moreover, this group frequently used stents with larger diameters (≥3 mm), longer lengths (≥35 mm), and employed multiple stent implantations (≥2 stents). In terms of lesion characteristics, the image-guided PCI group showed higher instances of multivessel disease (n = 1,174 [55.0%] vs n = 3,501 [49.1%]; p < 0.001) and complex left main vessel disease (n = 175 [8.2%] vs n = 183 [2.6%]; p < 0.001).

The PS matching results indicated adequacy based on standardized mean differences (SMD) in all clinical variables. Patient characteristics, such as age, sex, comorbidities, and lesion and procedure characteristics, were statistically similar between the two groups in this matched cohort (Table 1).

**Table 1:** Baseline characteristics of the unmatched and PS-matched cohort.

	Unmatched Cohort			PS-matched Cohort		
Variables	Angiography-guided PCI (n = 7,137)	Image-guided PCI (n = 2,134)	P value	Angiography-guided PCI (n = 2,089)	Image-guided PCI (n = 2,089)	P value	SMD
**Patient characteristics**							
Age, mean ± SD	63.29 ± 12.39	62.23 ± 12.10	<0.001	62.18 (12.28)	62.29 (12.12)	0.774	0.009
Sex, Male (%)	5,347 (74.9)	1,693 (79.3)	<0.001	1,657 (79.3)	1,653 (79.1)	0.909	0.005
BMI, mean ± SD (kg/ m^2^)	24.09 ± 3.20	24.17 ± 3.30	0.356	24.17 ±3.19	24.16 ±3.30	0.912	0.003
Killip class ≥ 3 (%)	472 (6.6)	124 (5.8)	0.202	108 (5.2)	121 (5.8)	0.415	0.027
Final diagnosis (%)			<0.001			0.901	0.005
NSTEMI	3,383 (47.4)	1,187 (55.6)		1,156 (55.3)	1,151 (55.1)		
STEMI	3,754 (52.6)	947 (44.4)		933 (44.7)	938 (44.9)		
Hypertension (%)	3,598 (50.4)	963 (45.1)	<0.001	913 (43.7)	946 (45.3)	0.319	0.032
Diabetes (%)	1,948 (27.3)	537 (25.2)	0.055	525 (25.1)	523 (25.0)	0.972	0.002
Dyslipidemia (%)	833 (11.7)	252 (11.8)	0.893	243 (11.6)	246 (11.8)	0.923	0.004
Previous MI (%)	408 (5.7)	123 (5.8)	0.977	140 (6.7)	118 (5.6)	0.177	0.044
Previous CVA (%)	434 (6.1)	104 (4.9)	0.041	86 (4.1)	99 (4.7)	0.367	0.03
Current smoker (%)	2,882 (40.4)	924 (43.3)	0.017	884 (42.3)	902 (43.2)	0.595	0.017
LVEF, mean ± SD (%)	52.12 ±10.44	53.14 ±9.82	<0.001	53.15±10.12	53.05±9.82	0.766	0.009
eGFR, mean ± SD (mL/min/1.73 m^2^)	84.25 ± 36.85	85.89 ± 36.86	0.072	86.08±32.11	85.67±36.86	0.703	0.012
Troponin I, mean ± SD (ng/mL)	47.02 ± 96.11	32.93 ±118.25	<0.001	36.35±105.41	33.42±119.46	0.401	0.026
CK-MB, mean ± SD (ng/mL)	109.81 ±145.84	113.12 ±133.76	0.349	116.77±160.95	114.19±134.17	0.574	0.017
Discharge medication (%)							
DAPT	7,122 (99.8)	2,126 (99.6)	0.274	2,084 (99.8)	2,082 (99.7)	0.773	0.018
Aspirin	7,130 (99.9)	2,133 (100.0)	0.774	2,088 (100.0)	2,088 (100.0)	>0.99	<0.001
P2Y12 inhibitors			<0.001			0.604	0.042
Clopidogrel	4,540 (63.6)	1,315 (61.6)		1,266 (60.6)	1,288 (61.7)		
Ticagrelor	1,576 (21.1)	596 (27.9)		579 (27.7)	579 (27.7)		
Prasugrel	1,013 (14.2)	216 (10.1)		240 (11.5)	216 (10.3)		
RAS inhibitors	5,981 (83.8)	1,713 (80.3)	0.001	1,680 (80.4)	1,681 (80.5)	>0.99	0.001
Beta-blocker	6,259 (87.7)	1,799 (84.3)	<0.001	1,752 (83.9)	1,762 (84.3)	0.703	0.013
Statin	6,796 (95.2)	2,062 (96.6)	0.007	2,028 (97.1)	2,018 (96.6)	0.426	0.027
**Procedural characteristics**							
Successful PCI (%)	7,110 (99.6)	2,123 (99.5)	0.498	2,076 (99.4)	2,078 (99.5)	0.838	0.013
Trans-radial approach (%)	2,683 (37.6)	920 (43.1)	<0.001	914 (43.8)	900 (43.1)	0.685	0.014
Usage of GP2b3a inhibitor (%)	989 (13.9)	389 (18.2)	<0.001	403 (19.3)	378 (18.1)	0.341	0.031
Thrombus aspiration (%)	1,763 (24.7)	468 (21.9)	0.009	475 (22.7)	464 (22.2)	0.711	0.013
Stent diameter ≥ 3mm (%)	5,122 (71.8)	1,673 (78.4)	<0.001	1,638 (78.4)	1,628 (77.9)	0.736	0.012
Stent length ≥ 35mm (%)	1,762 (24.7)	637 (29.9)	<0.001	605 (29.0)	626 (30.0)	0.497	0.022
Number of stents ≥ 2 (%)	2,293 (32.1)	837 (39.2)	<0.001	820 (39.3)	813 (38.9)	0.849	0.007
Stent type (%)			0.001			0.759	0.034
Biolimus	1,408 (19.7)	362 (17.0)		383 (18.3)	360 (17.2)		
Everolimus	3,614 (50.6)	1,118 (52.4)		1,074 (51.4)	1,099 (52.6)		
Zotarolimus	1,692 (23.7)	554 (26.0)		537 (25.7)	530 (25.4)		
etc	423 (5.9)	100 (4.7)		95 (4.5)	100 (4.8)		
**Lesion characteristics**							
Number of vessel disease (%)			<0.001			0.952	0.026
Left main disease (simple)	12 (0.2)	25 (1.2)		10 (0.5)	12 (0.6)		
Left main disease (complex)	183 (2.6)	175 (8.2)		143 (6.8)	143 (6.8)		
One-vessel disease	3,636 (50.9)	960 (45.0)		960 (46.0)	960 (46.0)		
Two-vessel disease	2,091 (29.3)	620 (29.1)		606 (29.0)	620 (29.7)		
Three-vessel disease	1,215 (17.0)	354 (16.6)		370 (17.7)	354 (16.9)		
Multivessel disease (%)	3,501 (49.1)	1,174 (55.0)	<0.001	1,129 (54.0)	1,129 (54.0)	>0.99	<0.001
Culprit vessel (%)			<0.001			0.922	0.022
Left main	76 (1.1)	99 (4.6)		62 (3.0)	69 (3.3)		
LAD	3,376 (47.3)	1,107 (51.9)		1,104 (52.8)	1,098 (52.6)		
LCX	1,226 (17.2)	340 (15.9)		331 (15.8)	337 (16.1)		
RCA	2,459 (34.5)	588 (27.6)		592 (28.3)	585 (28.0)		
ACC/AHA B2/C lesion (%)	6,196 (86.8)	1,822 (85.4)	0.096	1,788 (85.6)	1,787 (85.5)	>0.99	0.001

Data are expressed as mean ± standard deviation (SD) or number (percent). MI, myocardial infarction; STEMI, ST segment elevation myocardial infarction; NSTEMI, non-ST segment elevation myocardial infarction; CVA, cerebrovascular accident; TIA, transient ischemic attack; RAS inhibitors, renin-angiotensin-system inhibitors; LAD, left anterior descending artery; LCX, left circumflex artery; LM, left main; RCA, right coronary artery; eGFR, estimated glomerular filtration rate (calculated using MDRD GFR equation); LVEF, left ventricular ejection fraction; CK-MB, creatine kinase-myocardial band; DAPT, dual antiplatelet agent; GP2b3a inhibitor, glycoprotein IIb/IIIa inhibitors.

Within the image-guided PCI group, the IVUS- and OCT-guided subgroups were statistically similar in mean age, sex, body mass index, clinical manifestation, incidence of diabetes, dyslipidemia, previous myocardial infarctions, and cardiac enzyme levels. The exceptions were a previous history of cerebrovascular accidents (n = 100 [5.2%] vs n = 4 [1.9%]; p = 0.046) and the use of P2Y12 inhibitors and beta-blockers (n = 1,597 [83.2%] vs n = 202 [94.0%]; p < 0.001). Furthermore, the initial eGFR was high in the OCT-guided PCI group (94.51 ± 26.29 mL/min/1.73 m^2^ vs 84.92 ± 37.75 mL/min/1.73 m^2^; p < 0.001).

Given the substantial disparity in patient numbers, PS matching at a ratio of 2 was also performed within the image-guided PCI group, resulting in no significant differences between the matched groups according to SMD (Table 2).

**Table 2:** Baseline characteristics of unmatched cohort and PS-matched cohort of image-guided PCI.

	Unmatched Cohort			PS-matched Cohort			
Variables	IVUS-guided PCI (n = 1919)	OCT-guided PCI (n = 215)	P value	IVUS-guided PCI (n = 419)	OCT-guided PCI (n = 214)	P value	SMD
**Patient characteristics**							
Age, mean ± SD	62.36±12.21	61.01±11.01	0.12	61.05±12.34	61.07±10.99	0.98	0.002
Sex, Male (%)	1,518 (79.1)	175 (81.4)	0.485	344 (82.1)	174 (81.3)	0.892	0.02
BMI, mean ± SD (kg/ m^2^)	24.18±3.33	24.07±2.94	0.632	24.01±3.22	24.07±2.95	0.817	0.02
Killip class ≥ 3 (%)	111 (5.8)	13 (6.0)	0.998	27 (6.4)	13 (6.1)	0.994	0.015
Final diagnosis (%)			0.477			0.966	0.011
NSTEMI	1,062 (55.3)	125 (58.1)		245 (58.5)	124 (57.9)		
STEMI	857 (44.7)	90 (41.9)		174 (41.5)	90 (42.1)		
Hypertension (%)	879 (45.8)	84 (39.1)	0.07	163 (38.9)	83 (38.8)	>0.99	0.002
Diabetes (%)	489 (25.5)	48 (22.3)	0.353	99 (23.6)	48 (22.4)	0.812	0.028
Dyslipidemia (%)	232 (12.1)	20 (9.3)	0.276	48 (11.5)	20 (9.3)	0.499	0.069
Previous MI (%)	114 (5.9)	9 (4.2)	0.372	21 (5.0)	9 (4.2)	0.8	0.038
Previous CVA (%)	100 (5.2)	4 (1.9)	0.046	9 (2.1)	4 (1.9)	>0.99	0.02
Current smoker (%)	819 (42.7)	105 (48.8)	0.098	214 (51.1)	105 (49.1)	0.694	0.04
LVEF, mean ± SD (%)	53.17±9.93	52.95±8.76	0.762	53.04±9.94	52.95±8.78	0.914	0.009
eGFR, mean ± SD (mL/min/1.73 m^2^)	84.92±37.75	94.51±26.29	<0.001	91.23±33.76	94.53±26.35	0.212	0.109
Troponin I, mean ± SD (ng/mL)	32.97±122.64	32.63±67.61	0.969	29.81±56.57	32.78±67.73	0.559	0.048
CK-MB, mean ± SD (ng/mL)	112.51±130.64	118.59±159.20	0.527	120.40±149.50	119.12±159.38	0.921	0.008
Discharge medication (%)							
DAPT	1,912 (99.6)	214 (99.5)	>0.99	417 (99.5)	213 (99.5)	>0.99	0.001
Aspirin	1,918 (99.9)	215 (100.0)	>0.99	419 (100.0)	214 (100.0)	>0.99	<0.001
P2Y12 inhibitors			<0.001			0.959	0.046
Clopidogrel	1,180 (61.5)	135 (62.8)		272 (64.9)	135 (63.1)		
Ticagrelor	560 (29.2)	36 (16.7)		70 (16.7)	36 (16.8)		
Prasugrel	173 (9.0)	43 (20.0)		75 (17.9)	42 (19.6)		
RAS inhibitors	1,536 (80.0)	177 (82.3)	0.479	342 (81.6)	177 (82.7)	0.82	0.028
Beta-blocker	1,597 (83.2)	202 (94.0)	<0.001	400 (95.5)	201 (93.9)	0.519	0.069
Statin	1,851 (96.5)	211 (98.1)	0.273	415 (99.0)	210 (98.1)	0.55	0.078
**Procedural characteristics**							
Successful PCI (%)	1,909 (99.5)	214 (99.5)	>0.99	418 (99.8)	213 (99.5)	>0.99	0.039
Trans-radial approach (%)	832 (43.4)	88 (40.9)	0.543	177 (42.2)	88 (41.1)	0.853	0.023
Usage of GP2b3a inhibitor (%)	371 (19.3)	18 (8.4)	<0.001	33 (7.9)	18 (8.4)	0.936	0.02
Thrombus aspiration (%)	424 (22.1)	44 (20.5)	0.645	91 (21.7)	44 (20.6)	0.815	0.028
Stent diameter ≥ 3mm (%)	1,515 (78.9)	158 (73.5)	0.079	309 (73.7)	158 (73.8)	>0.99	0.002
Stent length ≥ 35mm (%)	595 (31.0)	42 (19.5)	0.001	89 (21.2)	42 (19.6)	0.711	0.04
Number of stents ≥ 2 (%)	770 (40.1)	67 (31.2)	0.013	134 (32.0)	67 (31.3)	0.935	0.014
Stent type (%)			<0.001			0.969	0.042
Biolimus	297 (15.5)	65 (30.2)		122 (29.1)	65 (30.4)		
Everolimus	1,036 (54.0)	82 (38.1)		158 (37.7)	82 (38.3)		
Zotarolimus	496 (25.8)	58 (27.0)		119 (28.4)	57 (26.6)		
etc	90 (4.7)	10 (4.7)		20 (4.8)	10 (4.7)		
**Lesion characteristics**							
Number of vessel disease (%)			0.002			0.702	0.101
Left main disease (simple)	25 (1.3)	0 (0.0)		0 (0)	0 (0)		
Left main disease (complex)	168 (8.8)	7 (3.3)		15 (3.6)	7 (3.3)		
One-vessel disease	843 (43.9)	117 (54.4)		212 (50.6)	116 (54.2)		
Two-vessel disease	557 (29.0)	63 (29.3)		142 (33.9)	63 (29.4)		
Three-vessel disease	326 (17.0)	28 (13.0)		50 (11.9)	28 (13.1)		
Multivessel disease (%)	1,076 (56.1)	98 (45.6)	0.004	207 (49.4)	98 (45.8)	0.438	0.072
Culprit vessel (%)			0.184			0.894	0.066
Left main	95 (5.0)	4 (1.9)		10 (2.4)	4 (1.9)		
LAD	996 (51.9)	111 (51.6)		218 (52.0)	111 (51.9)		
LCX	306 (15.9)	34 (15.8)		73 (17.4)	34 (15.9)		
RCA	522 (27.2)	66 (30.7)		118 (28.2)	65 (30.4)		
ACC/AHA B2/C lesion (%)	1,644 (85.7)	178 (82.8)	0.302	351 (83.8)	178 (83.2)	0.938	0.016

Data are expressed as mean ± standard deviation (SD) or number (percent). MI, myocardial infarction; STEMI, ST segment elevation myocardial infarction; NSTEMI, non-ST segment elevation myocardial infarction; CVA, cerebrovascular accident; TIA, transient ischemic attack; RAS inhibitors, renin-angiotensin-system inhibitors; LAD, left anterior descending artery; LCX, left circumflex artery; LM, left main; RCA, right coronary artery; eGFR, estimated glomerular filtration rate (calculated using MDRD GFR equation); LVEF, left ventricular ejection fraction; CK-MB, creatine kinase-myocardial band; DAPT, dual antiplatelet agent; GP2b3a inhibitor, glycoprotein IIb/IIIa inhibitors.

### Clinical outcomes

**Fig 2:**
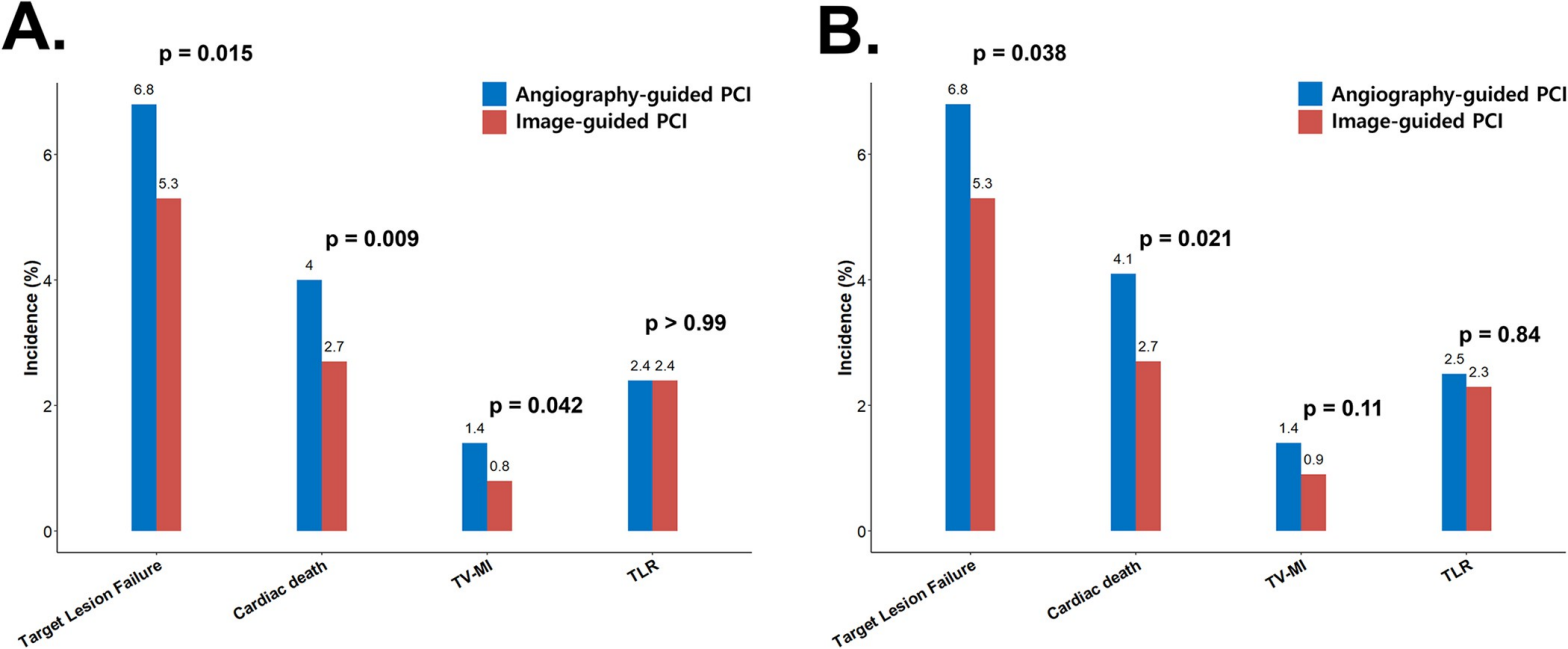
Clinical outcomes between image-guided and angiography-guided PCI (A) Unmatched cohort. (B) PS-matched cohort. TV-MI, target vessel myocardial infarction; TLR, ischemia-driven target vessel revascularization; PCI, percutaneous coronary intervention.

The main study outcomes are summarized in Fig 2 and Table 3. In the overall population, the incidence of TLF was significantly lower in the image-guided PCI group than in the angiography-guided PCI (n = 485 [6.8%] vs n = 113 [5.3%], p = 0.015), primarily owing to a reduction in cardiac deaths (n = 283 [4.0%] vs n = 58 [2.7%], p = 0.009) and target vessel myocardial infarctions (n = 103 [1.4%] vs n = 18 [0.8%], p = 0.042). Following PS matching, the incidence of TLF remained statistically lower in the image-guided PCI group than in the angiography-guided PCI (n = 143 [6.8%] vs n = 110 [5.3%], p = 0.038), mainly driven by a decrease in cardiac deaths (n = 85 [4.1%] vs n = 57 [2.7%], p = 0.021). According to the stratified Cox proportional hazard model in the matched cohort, image-guided PCI was associated with a significant reduction in TLF (HR: 0.76, 95% CI: 0.59–0.98; p = 0.035) and cardiac death (HR: 0.67, 95% CI: 0.48–0.92; p = 0.016) (Table 4).

**Table 3:** Clinical outcomes between image-guided and angiography-guided PCI.

**(A) Unmatched cohort**			
Outcomes	**Angiography-guided PCI (n = 7137)**	**Image-guided PCI** **(n = 2134)**	**P value**
Target lesion failure (%)	485 (6.8)	113 (5.3)	0.015
All cardiac deaths (%)	283 (4.0)	58 (2.7)	0.009
Target vessel myocardial infarction (%)	103 (1.4)	18 (0.8)	0.042
Ischemia-driven target vessel revascularization (%)	171 (2.4)	51 (2.4)	>0.99
**(B) PS-matched cohort**			
Outcomes	**Angiography-guided PCI (n = 2089)**	**Image-guided PCI** **(n = 2089)**	**P value**
Target lesion failure (%)	143 (6.8)	110 (5.3)	0.038
All cardiac deaths (%)	85 (4.1)	57 (2.7)	0.021
Target vessel myocardial infarction (%)	30 (1.4)	18 (0.9)	0.11
Ischemia-driven target vessel revascularization (%)	52 (2.5)	49 (2.3)	0.84

Data are expressed as mean ± standard deviation (SD) or number (percent).

**Table 4:** Comparing image-guided and angiography-Guided PCI using a stratified Cox proportional hazards model in the PS-matched cohort.

Outcomes	Hazard Ratio	95% Confidence Interval	P value
Target lesion failure (%)	0.76	0.59–0.98	0.035
All cardiac deaths (%)	0.67	0.48–0.92	0.016
Target vessel myocardial infarction (%)	0.59	0.33–1.07	0.08
Ischemia driven target vessel revascularization (%)	0.94	0.63–1.39	0.769

**Fig 3:**
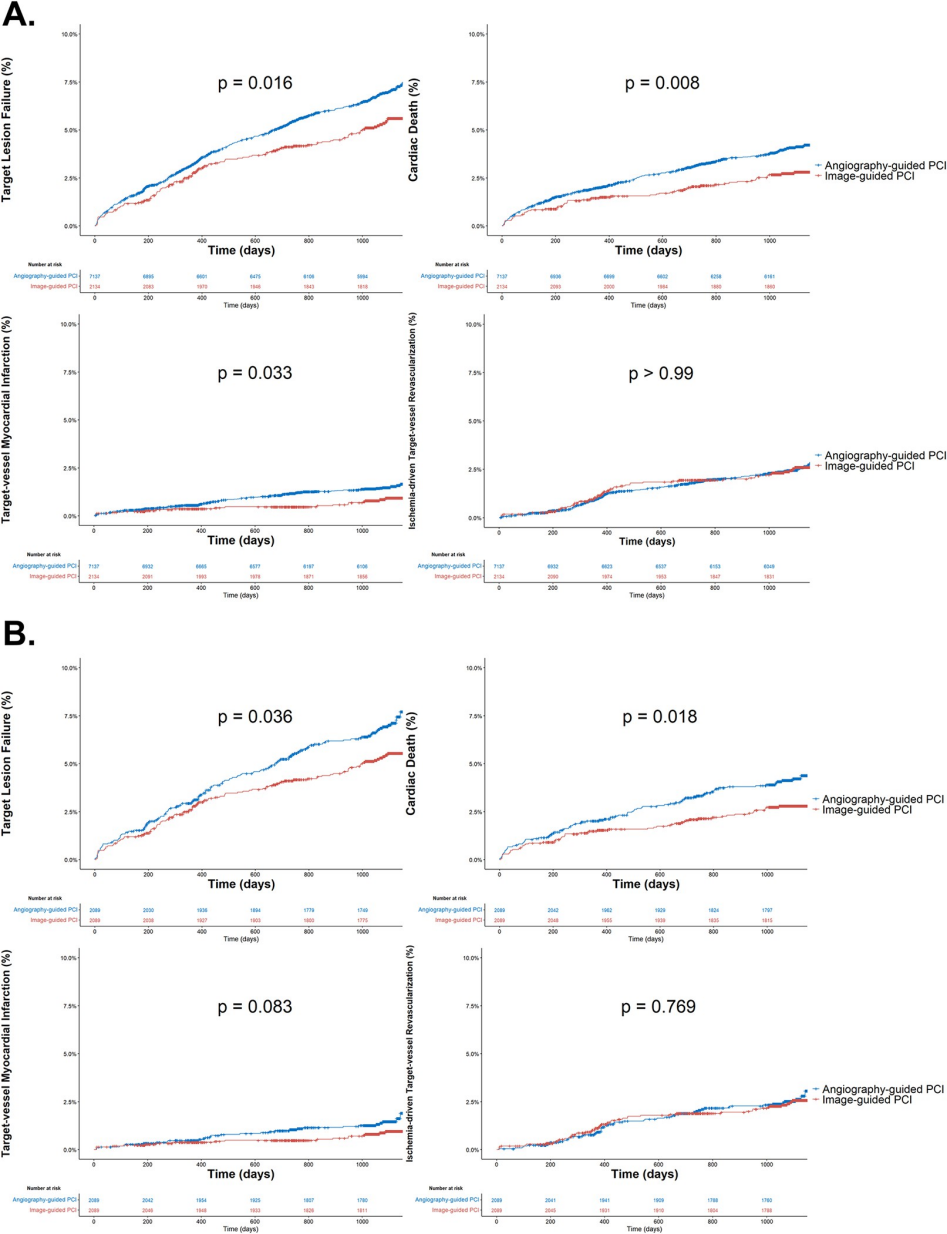
Kaplan–Meier survival analysis comparing outcomes between image-guided and angiography-guided PCI (A) Unmatched cohort. (B) PS-matched cohort. PCI, percutaneous coronary intervention.

The survival analysis, depicted using Kaplan–Meier curves, is presented in Fig 3. In the unmatched population, the 3-year cumulative incidences of TLF, cardiac death, and target vessel myocardial infarction were statistically lower in the image-guided PCI group than in the angiography-guided PCI (6.8% vs 5.3%, log-rank p = 0.016; 4.0% vs 2.7%, log-rank p = 0.008; 1.4% vs 0.8%, log-rank p = 0.033). The cumulative incidences of TLF and cardiac deaths remained significantly low in the image-guided PCI group (6.8% vs 5.3%, log-rank p = 0.036; 4.1% vs 2.7%, log-rank p = 0.018) following PS matching, except target vessel myocardial infarction (1.4% vs 0.9%, log-rank p = 0.083).

**Fig 4:**
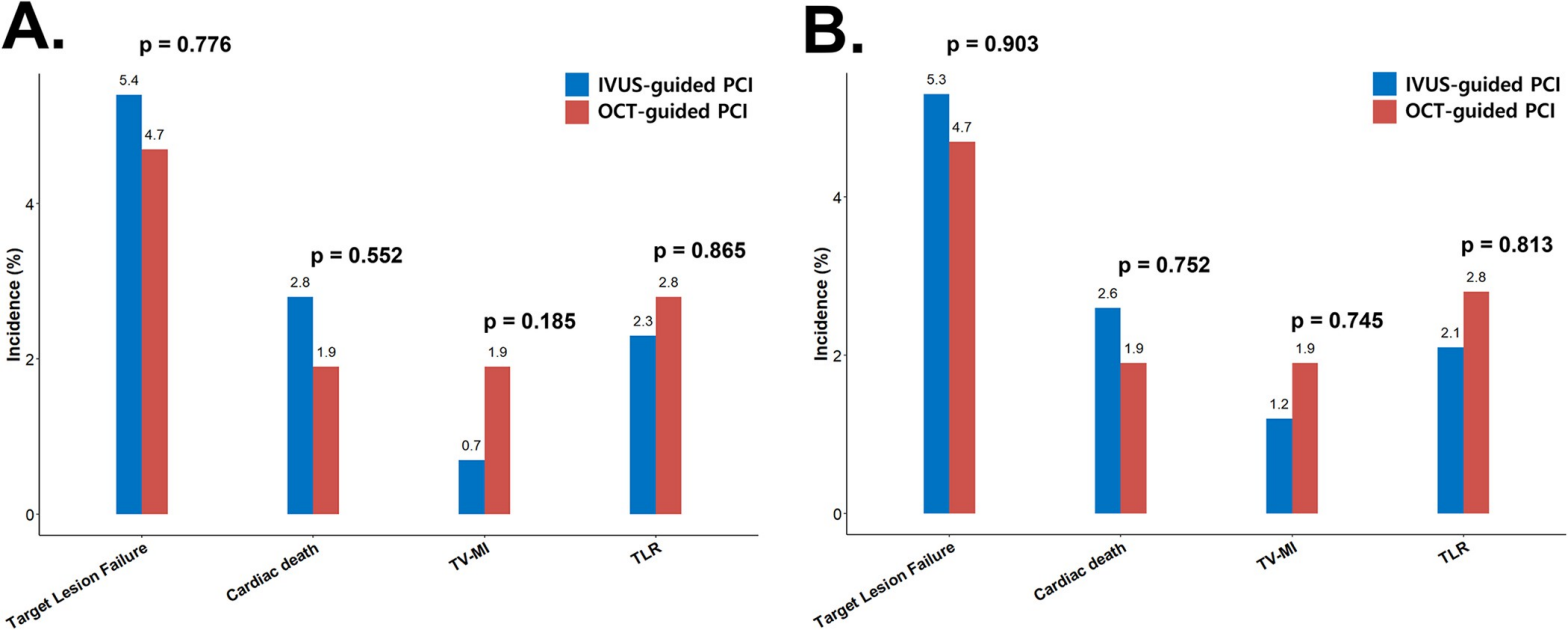
Clinical outcomes between IVUS- and OCT-guided PCI (A) Unmatched cohort. (B) PS-matched cohort. TV-MI, target vessel myocardial infarction; TLR, ischemia-driven target vessel revascularization; PCI, percutaneous coronary intervention; IVUS, intravascular ultrasound; OCT, optical coherence tomography.

**Fig 5:**
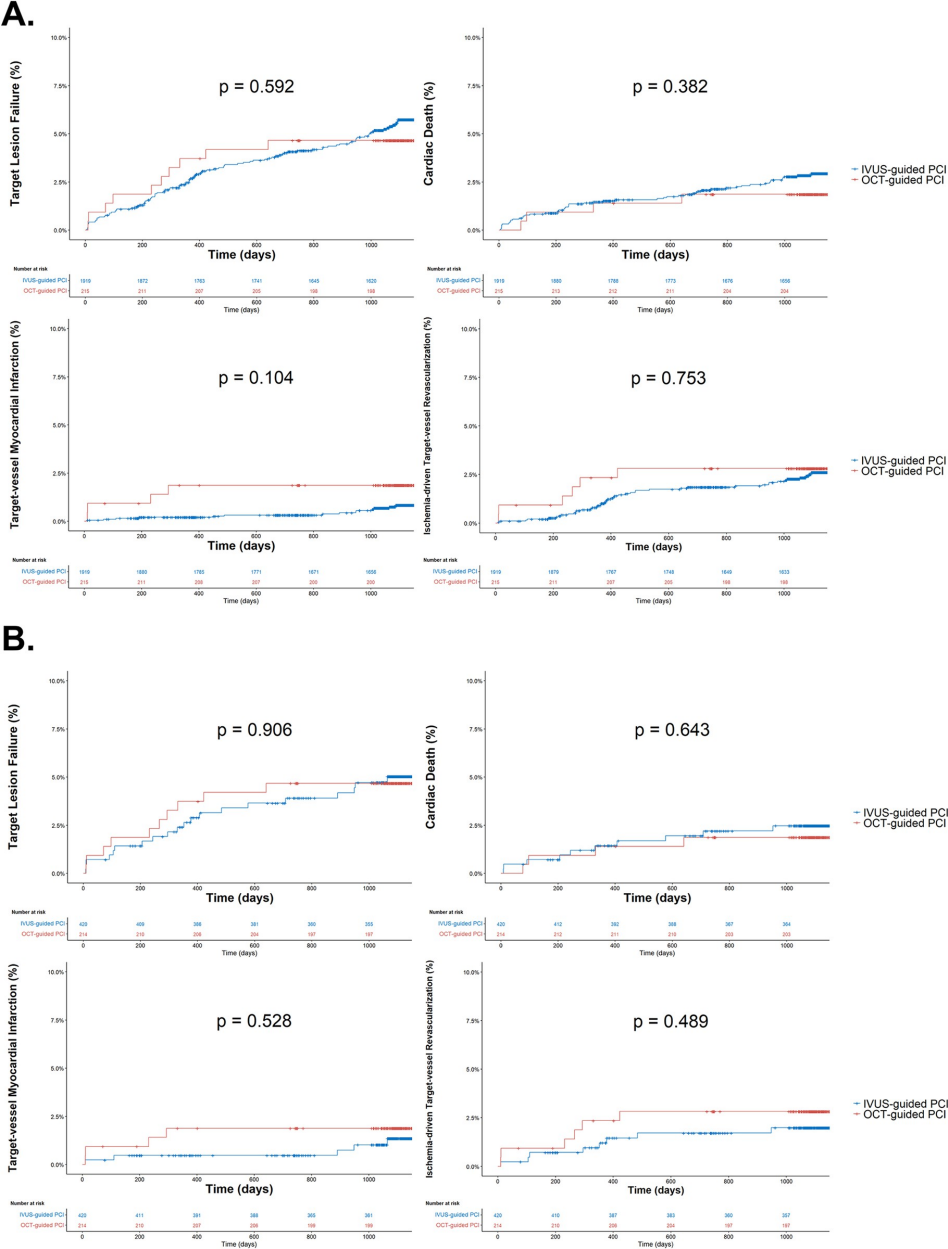
Kaplan–Meier survival analysis comparing outcomes between IVUS-guided and OCT-guided PCI (A) Unmatched cohort. (B) PS-matched cohort. PCI, percutaneous coronary intervention; IVUS, intravascular ultrasound; OCT, optical coherence tomography.

In the subgroup analysis, clinical outcomes were compared between the IVUS- and OCT-guided PCI groups (Fig 4 and Table 5). All outcomes, including TLF, cardiac death, treated vessel myocardial infarction, and ischemia-driven target vessel revascularization, were statistically similar between the OCT- and IVUS-guided PCI groups in the unmatched and PS-matched cohorts. Furthermore, in the survival analysis, clinical outcomes between the IVUS- and OCT-guided PCI groups were similar, both before and after PS matching, as illustrated in Fig 5.

**Table 5:** Clinical outcomes between IVUS-guided and OCT-guided PCI.

**(A) Unmatched cohort**			
Outcomes	**IVUS-guided PCI** **(n = 1919)**	**OCT-guided PCI** **(n = 215)**	**P value**
Target lesion failure (%)	103 (5.4)	10 (4.7)	0.776
All cardiac death (%)	54 (2.8)	4 (1.9)	0.552
Target vessel myocardial infarction (%)	14 (0.7)	4 (1.9)	0.185
Ischemia-driven target vessel revascularization (%)	45 (2.3)	6 (2.8)	0.865
**(B) PS-matched cohort**			
Outcomes	**IVUS-guided PCI** **(n = 419)**	**OCT-guided PCI** **(n = 214)**	**P value**
Target lesion failure (%)	22 (5.3)	10 (4.7)	0.903
All cardiac death (%)	11 (2.6)	4 (1.9)	0.752
Target vessel myocardial infarction (%)	5 (1.2)	4 (1.9)	0.745
Ischemia-driven target vessel revascularization (%)	9 (2.1)	6 (2.8)	0.813

Data are expressed as mean ± standard deviation (SD) or number (percent).

## Discussion

In this study, we found that image-guided PCI in AMI resulted in more favorable clinical outcomes compared with angiography-guided PCI, based on data from a large-scale, multicenter, nationwide registry. Additional clinical implications of our study are as follows: First, our study included only AMI patients and was followed up for 3 years. Several studies have explored the importance of image guidance in coronary interventions. However, contrary to previous research that included a mix of conditions like chronic coronary syndrome and acute coronary syndrome, our study focused on a specific subset of patients with AMI. Furthermore, the follow-up duration of this study was considerably longer than that of previous studies. Second, the estimated frequency of IVUS or OCT use in patients with AMI in Korea was 23.0%, which is consistent with the IVUS usage rate reported in another Korean registry regarding myocardial infarction and is considerably higher than the IVUS usage rate in the United States (3.17%) [11,12]. Third, it has been well established recently that OCT correlates effectively with the IVUS measurements when investigating lesion characteristics [1,13,14]. Despite the similar capabilities of OCT, most previous research compared IVUS-guided PCI with angiography-guided PCI [8,15,16]. In this study, however, we have shown the favorable outcomes associated with both IVUS and OCT in AMI. Additionally, the subgroup analysis regarding clinical outcomes within the image-guided PCI group suggests that OCT is comparable to IVUS and can be used as an alternative to IVUS for stent optimization in patients with AMI.

This study has several limitations. First, it was a retrospective cohort study based on registry data. Although we used the PS-matched cohort to analyze between-group differences, the existence of confounding factors cannot be ruled out. Furthermore, the decision to use IVUS, OCT, or angiography only was predominantly made by treating cardiologists and centers, potentially altering the results and rationale of the treating interventionists. Moreover, the absence of definitive criteria for image-guided stent optimization indicates that the experience of the operator and the policies of the institution may influence clinical outcomes. Finally, a substantial disparity in the number of patients between the IVUS and OCT groups was present.

## Conclusion

This large-scale, nationwide registry reveals that image-guided PCI, including IVUS and OCT, is associated with favorable clinical outcomes in patients with AMI. Additionally, OCT-guided PCI is not inferior to IVUS-guided PCI and can be used as an alternative in patients with AMI.
